# Enrichment of Double RUNX1 Mutations in Acute Leukemias of Ambiguous Lineage

**DOI:** 10.3389/fonc.2021.726637

**Published:** 2021-08-31

**Authors:** Gabriele Merati, Marianna Rossi, Anna Gallì, Elisa Roncoroni, Silvia Zibellini, Ettore Rizzo, Daniela Pietra, Cristina Picone, Barbara Rocca, Claudia Patricia Tobar Cabrera, Eleonora Gelli, Eugenio Santacroce, Luca Arcaini, Patrizia Zappasodi

**Affiliations:** ^1^Division of Hematology, Fondazione IRCCS Policlinico San Matteo, Pavia, Italy; ^2^enGenome, Pavia, Italy; ^3^Department of Molecular Medicine, University of Pavia, Pavia, Italy

**Keywords:** Runx1, acute undifferentiated leukaemia, myeloid genes, acute leukemia of ambiguous lineage, double mutations

## Abstract

Acute leukemia of ambiguous lineage (ALAL) is a rare type of leukemia and represents an unmet clinical need. In fact, due to heterogeneity, substantial rarity and absence of clinical trials, there are no therapeutic guidelines available. We investigated the genetic basis of 10 cases of ALAL diagnosed at our centre from 2008 and 2020, through a targeted myeloid and lymphoid sequencing approach. We show that this rare group of acute leukemias is enriched in myeloid-gene mutations. In particular we found that RUNX1 mutations, which have been found double mutated in 40% of patients and tend to involve both alleles, are associated with an undifferentiated phenotype and with lineage ambiguity. Furthermore, because this feature is typical of acute myeloid leukemia with minimal differentiation, we believe that our data strengthen the idea that acute leukemia with ambiguous lineage, especially those with an undifferentiated phenotype, might be genetically more closer to acute myeloid leukemia rather than acute lymphoblastic leukemia. These data enrich the knowledge on the genetic basis of ALAL and could have clinical implications as an acute myeloid leukemia (AML) – oriented chemotherapeutic approach might be more appropriate.

## Introduction

Acute leukemia of ambiguous lineage is a group of rare leukaemia with mixed features of lymphoblastic and myeloid lineage, which represents <4% of all acute leukemias (1).

The 2016 WHO classification of myeloid and lymphoid neoplasms recognizes seven entities under the category of acute leukemias of ambiguous lineage (ALAL), including mixed phenotype acute leukemia (MPAL) with t(9;22)(q34.1;q11.2) *BCR-ABL1*, MPAL with t(v;11q23.3) KMT2A rearranged, MPAL B/myeloid NOS, MPAL T/myeloid NOS, MPAL not otherwise specified (NOS) rare types, acute undifferentiated leukemia (AUL), and acute leukemia of ambiguous lineage, not otherwise specified (ALAL NOS).

Mixed phenotype acute leukemia (MPAL) is characterized in most cases by a single population of blast cells that express both immunophenotypic features of myeloid origin, as MPO, and markers of B- or T-cell lineage (cCD3 for T cell or CD19 plus CD22 and/or CD79a for B-cell commitment).

MPALs with recurrent cytogenetic abnormalities (BCR-ABL1 and KMT2A rearrangements) represent distinct entities in the last WHO classification; MPALs without these anomalies have been termed MPAL B/myeloid and T/myeloid NOS.

Acute undifferentiated leukemia (AUL) is the rarest type of ALAL. By definition, AUL usually expresses no more than one marker for B-cell, T-cell, or myeloid lineage. Blasts often express the early hematopoietic-associated antigens CD34, CD38, human leukocyte antigen (HLA)-DR, and may be positive for TdT. Attention must be paid in differential diagnosis between AUL and acute myeloid leukemia (AML) with minimal differentiation, which grossly corresponds to the previous M0 by the French–American–British (FAB) classification.

ALALs that do not fulfil the diagnostic criteria for MPAL or AUL should be classified as ALAL NOS.

The immunophenotypic heterogeneity of ALAL likely reflects a heterogeneous mutational profile. Literature data on the mutational landscape of ALAL are sparse and controversial and have been conducted on small cohorts, mainly composed of MPAL (2–4). Overall, they suggest that MPAL share common genetic features with acute lymphoblastic leukemia (ALL). On the other hand, sporadic genetic data on AUL seem to suggest that they are more similar to AML and share a mutational and gene expression profile with AML (3).

An increased biological knowledge on ALAL could have clinical implications such as the choice of an appropriate therapeutic approach: in fact, the treatment of ALAL remains a matter of debate, and it is not clear whether patients benefit from ALL- or AML-oriented chemotherapeutic regimens (5, 6).

Therefore, there is a great need of further studies to better characterize the biology of this group of rare leukemias, which is the first step for setting an effective treatment strategy that is still an unmet clinical need.

## Methods

To address the need of a better biological definition of ALAL, we identified 12 patients who received a diagnosis of ALAL between 2008 and 2020 at the Division of Hematology, Fondazione IRCCS Policlinico San Matteo of Pavia.

All diagnoses have been carefully reviewed according to (1) criteria. The review process was conducted with particular attention to MPALs because the WHO 2016 classification states that some well-defined myeloid leukemia entities have immunophenotypic features that suggest that they be classified as B/myeloid or T/myeloid leukemia. These cases should be classified within their main WHO category, with a secondary annotation that they present a mixed phenotype (1). Therefore, ALAL remains mainly a diagnosis of exclusion. As a result, patients 1 and 8 have been reclassified from MPALs to the AML with myelodysplasia-related changes (AML-MRC) category.

We conducted a mutational analysis on bone marrow samples on the 10 patients with confirmed diagnosis, by using a targeted sequencing approach with a 54 myeloid and a 138 lymphoid gene panels on a HiSeq2500 Illumina (**Supplemental Table 1**). Detailed methods used with each gene panel have been previously published (7, 8). Bone marrow mononuclear cells of patients at diagnosis were analyzed. All variants have been confirmed by whole exome sequencing (WES).

When available, to state the germline or somatic nature of RUNX1 mutations mesenchymal stem cells cultured from bone marrow, skin fibroblasts or cells isolated from buccal rinse were sequenced. The relapse sample of patient 9 was also sequenced.

Copy-number variants (CNVs) calling was performed with ExomeDepth (9), an algorithm that uses a read depth strategy to call CNVs from exome or targeted sequencing data. This approach aims to compare each case with a matched aggregate reference set that is created combining the sequencing results of the study.

In a subset of cases with RUNX1 double mutations, we explored the reads generated from DNA or RNA libraries spanning both positions in order to determine if mutations involved one allele (*cis*) or both alleles (*trans*). The reads presenting only one variant were considered in *trans*, and the reads presenting both variants were considered in *cis.* The fraction of reads with *cis* configuration and the fraction with *trans* configuration were then calculated by dividing the number of in *cis* reads and in *trans* reads by the number of total reads. DNA libraries were prepared using Illumina TruSight Myeloid Panel.

For patients with RUNX1 mutations not covered by unique DNA sequencing reads (i.e., >250 bp distance), an *ad hoc* procedure was developed for the analysis of RNA. Briefly, specific primers with overhang adapter sequences were designed to amplify the region encompassing the two RUNX1 mutations. Illumina sequencing adapters and dual-index barcodes were added to the amplicon target through a limited cycle of PCR. Libraries were normalized, pooled, and loaded onto a 500-cycle MiSeq sequencer flow cell.

## Results

Our study cohort resulting from the diagnostic review consists of five AUL, two MPAL B/myeloid NOS, one MPAL T/myeloid NOS, and two ALAL NOS (**Table 1**); the median age at diagnosis was 64.5 years (range, 26–73).

**Table 1:** Flow cytometry and cytogenetic features of the ALAL cohort.

Patient	Diagnosis review (WHO 2016)	Age at diagnosis (year)	Flow Cytometry	Cytogenetic analysis
Pt 2	AUL	66	90% blasts: **CD34+,** CD117−, MPO−, **Tdt+**, **CD13dim**, **CD38+, DR+**, CD19−, CD79a−, CD2−, cCD3−, CD3, CD4−, CD5−, CD7−, CD8−, CD10−, CD14−, CD16−, CD19−, CD20−, CD11b−, CD11c−, CD33−, CD14−, CD15−, CD41−, CD56−, CD61−	46 XY
				FISH for 5q31/5p15,7q31/cen7, KMT2A, t(8;21) normal
Pt 3	ALAL NOS	71	57% blasts: **CD34+, CD117+**, MPO−, **Tdt+, CD7+, CD5+, CD99+, CD79a+, CD38+, CD33dim**, CD10−, CD19−, CD20−, cyCD22−, FMC7−, CD1a−, CD2−, CD3−, cCD3−, CD4−, CD8−, CD11c−, CD13−, CD15−, CD16−, CD56−	46 XX
				FISH for 5q31/5p15,7q31/cen7, KMT2A, t(8;21) normal
Pt 4	ALAL NOS	46	50% blasts: **CD34+**, CD117−, MPO−, Tdt−, **CD13+**, **CD33+**, **CD5+, CD7+, DR+, CD10+**, CD19−, CD20, cyCD22−, cyCD79a−, CD2−, CD3−, cyCD3−, CD4−, CD8−, CD11b−, CD11c−, CD14−, CD15−, CD16−, CD56−	46, XX, t(2;3)(p21;q26), del (6q)(q23), t(12);?(p13);?, ins (12);?(p13);?
				FISH for 5q31/5p15,7q31/cen7, KMT2A, t(8;21) normal, ETV6 and MECOM rearranged with unknown partner genes
Pt 5	AUL	72	27% blasts: **CD34+**, CD117−, MPO−, **Tdt+, CD13+, CD38+**, CD33−, **CD99+, DR+**, CD10−, CD19−, CD20−, cyCD22−, cyCD79a−, CD1a−, CD2−, CD3−, cyCD3−, CD4−, CD5−, CD7−, CD8−, CD11b−, CD11c−, CD14−, CD15−, CD16−, CD41−, CD61−, CD71−, CD25−, CD56−	48, XX, −14, + mar1, + dup(mar1), + mar2
				FISH for 5q31/5p15,7q31/cen7, KMT2A, t(8;21) normal
Pt 6	AUL	72	80% blasts: **CD34+, CD117+**, MPO−, Tdt−, **DR+, CD38+**, CD33−, CD13−, CD10−, CD19−, CD20−, cyCD22−, cyCD79a−, CD2−, CD3−, cyCD3−, CD4−, CD5−, CD7−, CD8−, CD11b−, CD11c−, CD14−, CD15−, CD16−, CD56−	46 XY
				FISH for 5q31/5p15,7q31/cen7, KMT2A, t(8;21) normal, BCR/ABL, rearrangements involving EV1, FGFR1, PDGFRB e JAK2 absent
Pt 7	MPAL B/myeloid	52	64% blasts: CD34−, CD117−, **MPO+, Tdt+, CD19+, CD79a+, CD10+, CD58+, CD38+, CD33dim**, CD10−, CD19−, cyCD22−, cyCD79a−, CD7−, cyCD3−, CD11c−, CD14−, CD16−	45, X
				FISH panel for 5q31/5p15,7q31/cen7, KMT2A, t(8;21) normal
Pt 9	MPAL B/myeloid bilinear	63	40% blasts: **CD34+, CD117+**, MPO−, **Tdt+, CD13+, CD33+, CD25+, DR+**. 28% blasts: **CD34+**, CD117−, MPO−, **Tdt+, CD19+, CD22+, CD25+**; CD10−, CD20−, cyCD79a−, CD2−, CD3−, cyCD3−, CD4−, CD5−, CD7−, CD8−, CD11b−, CD11c−, CD14−, CD15−, CD16−, CD56−	46, XY
Pt 10	MPAL T/myeloid	45	78% blasts: **CD34+, CD117+, MPO+**, Tdt−, **CD13+, CD33+, CD7+, CD5+, cyCD3+, CD3+**, DR−, CD2−, CD4−, CD8−, CD11b−, CD11c−, CD14−, CD15−, CD16−, CD25−, CD56−	Hyperdiploidy
Pt 11	AUL	73	65% blasts: **CD34+**, CD19−, **CD10dim**, **Tdt+,** CD20−, MPO−, **CD38+, CD117+**, CD13−, CD33−, CD3−, cyCD3−, CD2−, CD5−, CD7−, **cyCD22dim**, cyCD79a−	46, XY
Pt 12	AUL	26	91% blasts: **CD34+**, CD19−, **CD10dim, Tdt+**, CD20−, MPO−, **CD38+/**−, CD117−, CD13−, CD33−, CD3−, cyCD3−, CD2−, CD7−/+(dim), CD5−, CD25−, **cyCD22dim**, cyCD79a−	46, XY

Bold values highlight positive flow cytometry markers.

Seven (3 AUL, 2 ALAL-NOS, and 2 MPAL) of 10 patients have been treated with intensive chemotherapy. Two patients were considered unfit for intensive treatment, and one patient was lost to follow-up after diagnosis. Both ALL- and AML-oriented chemotherapeutic regimens showed some efficacy to induce complete remissions, supporting the ambiguous nature of disease. Overall, five patients achieved complete remission, and four proceeded to allogeneic bone marrow transplant (**Supplemental Table 2**).

The most frequently mutated genes within the myeloid panel were NRAS (40%; 4/10 patients), RUNX1 (40%; 4/10), ASXL1 (30%; 3/10), DNMT3A (20%; 2/10), BCOR (20%; 2/10), EZH2 (20%; 2/10), and U2AF1 (20%; 2/10). The only recurrently mutated lymphoid gene was KMT2C (25%, 2/8), but mutations within this gene were mainly subclonal (**Supplemental Table 3**). **Table 2** summarizes the mutational profile of the 10 ALAL patients.

**Table 2:** Mutational profile of the 10 patients with acute leukemia of ambiguous lineage.

	**Pt 2**	**Pt 5**	**Pt 6**	**Pt 11**	**Pt 12**	**Pt 7**	**Pt 9**	**Pt 10**	**Pt 3**	**Pt 4**	
**Diagnosis**	**AUL**	**AUL**	**AUL**	**AUL**	**AUL**	**MPAL B/M**	**MPAL B/M**	**MPAL T/M**	**ALAL NOS**	**ALAL NOS**	
**ASXL1**											Chromatin regulation
**BCOR**											
**EZH2**											
**KDM6A**											
**DNMT3A**											DNA methilation
**IDH2**											
**FLT3**											RTK-RAS signaling
**KRAS**											
**NRAS**											
**PTPN11**											
**SETBP1**											
**PHF6**											Transcription factor
**IKZF1**											
**RUNX1**											
**SF3B1**											RNA splicing
**SRSF2**											
**U2AF1**											
**ARID1A**											Chromatin regulation
**ATM**											Cell cycle apoptosis
**FGFR3**											RTK-RAS signaling
**KMT2C**											DNA methylation
**KMT2D**											
**NOTCH1**											NOTCH pathway
**PDE4DIP**											Microtubule assembly

We report only oncogenic and likely oncogenic single nucleotide variants and small indels identified with the 54 myeloid gene and 138 lymphoid gene panels. The green colored boxes indicate that patients 11 and 12 have not been studied with the lymphoid panel. The violet-colored boxes identify myeloid gene mutations. Orange-colored boxed identify lymphoid gene mutations.

The median number of mutations in myeloid genes [3; interquartile range (IQR), 1–4] was superior compared to the lymphoid ones (1; IQR, 0–19) (**Supplemental Figure 1**).

We then focused our attention on the RUNX1 gene, which is a well-known regulator of hematopoiesis and is essential for the development of both lymphoid and myeloid lineages (10).

All four RUNX1-mutated cases presented two mutations, mainly of founding type (**Table 3**): three AUL patients (patients 2, 11, and 12) and one patient (patient 9) with a MPAL B/myeloid bilinear NOS whose immunophenotype profile was a mixture of an AML with minimal differentiation and a B-cell acute lymphoblastic leukemia (B-ALL) (**Table 1**). Patients 2, 9, and 12 presented high RUNX1 variant allele frequency (VAF), suggesting that all variants were clonal (**Table 3**). Patient 11 was the only one who presented oncogenic variants with low allelic burdens, including RUNX1, and this has been addressed to hemodilution of the bone marrow aspirate.

**Table 3:** Pattern of RUNX1 mutations in three AUL patients and one MPAL B/myeloid.

Patient	Gene	Function	Chr	C.	P.	AB	Exon	WT	MT	Allele configuration P.	COSMIC ID	FATHMM pathogenic score
2	RUNX1	Stopgain SNV	21	c.777dupT	p.N260_P261delinsX	42.00	7	–	A	p.[F163Y][N260_P261delinsX]	NA	NA
	RUNX1	Missense	21	c.T488A	p.F163Y	43.30	5	A	T		COSM26023	0.99
9	RUNX1	Frameshift deletion	21	c.492delC	p.V164fs	26.58	5	G	–	NA	NA	NA
	RUNX1	Missense	21	c.A314G	p.H105R	37.40	4	T	C		NA	NA
11	RUNX1	Nonsense	21	c.427T	p.E143X	4.00	5	C	A	p.[E143X;p.A147_T148insRDA]	NA	NA
	RUNX1	Inframe insertion	21	c.442_443insGAGATGCTA	p.A147_T148insRDA	5.30	5	–	TAGCATCTC		NA	NA
12	RUNX1	Frameshift	21	c.382_383insAGAG	p.T128KfsX11	45.90	5	–	CTCT	p.[T128KfsX11];[p.R166G]	NA	NA
	RUNX1	Missense	21	c.496C>G	p.R166G	43.40	5	G	C		COSM24742	0.88

All four RUNX1 mutated patients present both a missense and a nonsense/frameshift variant. All variants were likely to be founding because allele burden (AB) are high in all patients except from patient 11 whose bone marrow sample was highly contaminated with peripheral blood (see also **Supplemental Table 3**).

In addition to SNVs and small insertions/deletions (indels), major RUNX1 deletions represent another possible mechanism of loss-of-function alteration. Therefore, we applied a copy number variant (CNV)-targeted approach for the identification of RUNX1 deletions, and we did not identify any larger deletions or insertions along the exons of the RUNX1 wild-type cases.

To investigate whether RUNX1 SNVs and indels were somatically acquired or inherited, we sequenced with the myeloid-gene panel the germline control tissues (bone marrow mesenchymal stem cells, buccal rinse cells, or skin fibroblasts) available in three RUNX1-mutated patients (patients 2, 9, and 12): all samples resulted wild type, thus confirming the somatic origin of these variants. Case 11 showed a low allele burden in the tumour sample (4% and 5%), and therefore, even in the absence of a control tissue, we can reasonably conclude that both mutations were somatically acquired.

To better understand the effect of RUNX1 double mutations, we studied the distribution of RUNX1 variants between the two alleles of the gene, as *cis* or *trans* configuration may have different biological implications on gene function and clinical phenotype.

In three samples (samples 2, 11, and 12), we were able to determine the allele configuration of RUNX1 composite mutations (**Table 3**).

The analysis of sequencing reads generated from DNA of the low VAF RUNX1 double mutated case (patient 11, **Table 3**) revealed a *cis* configuration of the two lesions.

The analysis of DNA and RNA sequencing reads in patients 12 and 2, respectively, showed a *trans* configuration of the two mutations; this allele configuration likely reduces the functional form of the gene, since both homologues are altered in nearly all cells.

In addition, the missense mutation identified in patient 2 (c.488T>A, p.F163Y) was predicted, by *in silico* splice site prediction tools (Human Splice Finder and Fruifly with score of 98.4 and 1, respectively), to induce the activation of a cryptic donor site that might affect pre‐mRNA processing. Accordingly with this prediction, the analysis of RNA reads showed the presence of a 23-nucleotide deletion in 49% of the reads resulting from a non-canonical splice site recognition (**Supplemental Figure 2**). This event induces a shift in the reading frame that predicts the termination of protein synthesis 41 codons downstream, thus resulting in a truncated protein. Sanger sequencing on complementary DNA (cDNA) confirmed next-generation sequencing (NGS) results (c.486-508del23, p.F163EfsX41). Moreover, the original c.488T>A and c.777dupT variants were found in the remaining 2% and 49% of the RNA reads, respectively. Therefore, wild-type reads were not detected, confirming that the RUNX1 variants are in *trans*, and substantially, no functional RNA was present.

Case 9 was also evaluated at relapse with the targeted myeloid panel. The mutational landscape at relapse showed overlapping with the sample at diagnosis except for the loss of the RUNX1 missense variant c.314A>G. Interestingly, the immunophenotype at relapse showed loss of the lymphoid markers Tdt, CD19, and CD22 and gain of MPO, thus corresponding to the immunophenotypic profile of an AML with minimal differentiation.

## Discussion

Our cohort of patients is limited and precludes the possibility of a significant statistical analysis. Despite this intrinsic limitation, the amount of mutations in myeloid genes was clearly exceeding the number of lymphoid ones, with statistical significance (**Supplemental Figure 1**). This imbalance could in part reflect the features of our cohort, which is particularly enriched in AUL, whereas the majority of studies on ALAL have been conducted on MPALs, and AULs were often underrepresented (4, 11, 12).

Overall, ALAL presents a heterogeneous genetic landscape, and both individual genes and pathways do not clearly cluster within the different disease subtypes (**Supplemental Figure 3**).

One of the most frequently mutated genes was RUNX1 (40%; 4/10), which was found double mutated in all cases. To gain insight into this particular feature, which was previously described in AML with minimal differentiation, we further characterized the RUNX1-mutated cases.

All RUNX1 SNV and indels resulted somatically acquired, excluding a familial predisposition leukemia with one inherited RUNX1 mutation, which is an increasingly recognized and often underdiagnosed condition (13). Furthermore, we excluded other major deletions/insertions that could affect RUNX1 gene.

Finally, we studied the *cis/trans* RUNX1 allele configuration in three patients (patient 9 could not be studied due to paucity of the sample) and revealed that two patients (patients 2 and 12) had mutations involving both alleles, while one patient (patient 11) presented both mutations on the same allele. Therefore, while for patients 2 and 12, we can reasonably speculate that there is substantially no functional RUNX1 protein left, patient 11 kept one RUNX1 allele intact.

Other factors can negatively affect RUNX1 protein function in the posttranscriptional phase, such as the dominant-negative effect, which is a well-known mechanism that has been described for some RUNX1 variants (14). Although it has not been demonstrated for patient 9, we cannot exclude that protein function be altered through other posttranscriptional events leading to a complete RUNX1 loss of function.

Patient 2 RUNX1 c.488T>A variant has been previously reported in the literature (15, 16) and has been interpreted as a missense mutation coding for p.F163Y. However, data about the mechanism of action of RUNX1 p.F163Y have not been provided, and we could not find sequences carrying c.486_508del23 in repositories of variants as Cosmic, ClinVar, HGMD, or Lovd.

Therefore, we gained insight into the oncogenic mechanism of c.488T>A variants, which, instead of a missense variant, results in a misplacing event that leads to a truncated protein. This underlines the importance to perform RNA analysis for the full evaluation of exonic mutations close to exon–intron border.

Patient 9 was evaluated at relapse where both the immunophenotypic properties and genetic landscape substantially changed. In particular, the disappearance of one RUNX1 variant at relapse was associated with the loss of lineage ambiguity (**Table 4**), and the immunophenotypic features at that point were coherent with an AML with minimal differentiation.

**Table 4:** Clonal evolution dynamics in patient 9.

Gene	Transcript	CCDS	EX	C.	P.	Allele burden—diagnosis	Allele burden—relapse
PHF6	NM_001015877	CCDS14639.1	9	c.C932G	p.A311G	0.8065	0.4013
RUNX1	NM_001754	CCDS13639.1	4	c.A314G	p.H105R	0.374	0
RUNX1	NM_001754	CCDS13639.1	5	c.492delC	p.V164fs	0.2658	0.3043
SF3B1	NM_012433	CCDS33356.1	13	c.G1774A	p.E592K	0.4347	0.2907
Immunophenotypic features of bone marrow blasts						40%: **CD34+, CD117+**, MPO−, **Tdt+, CD13+, CD33+, CD25+, DR+**. 28%: **CD34+,** CD117−, MPO−, **Tdt+, CD19+, CD22+, CD25+**; CD10−, CD20−, cyCD79a−, CD2−, CD3−, cyCD3−, CD4−, CD5−, CD7−, CD8−, CD11b−, CD11c−, CD14−, CD15−, CD16−, CD56−	15%: **CD34+, CD117+, MPO+, CD13+, CD33+, CD15+, DR+, CD38+**, Tdt−, CD19−, CD20−, CD22−, CD22cy−, CD79a−, CD2−, cyCD3−, CD7−, CD123−, CD10−, CD25−, CD56−, CD11b−

The double RUNX1 mutant genotype at diagnosis confers lineage ambiguity (MPAL B/myeloid NOS), whereas the disappearance of one RUNX1 variant at relapse gives rise to an AML phenotype.

Bold values highlight positive flow cytometry markers.

Taken together, these data suggest that the complete loss of function of RUNX1 correlates with the lack of expression of lineage-defining markers and contributes to lineage ambiguity.

Double RUNX1 mutations have been previously reported to be associated with AML with minimal differentiation (14), and RUNX1 mutations have been described in AUL (17) in up to 40% of cases, consistently with our results. Our data enrich previous knowledge on AUL, suggesting that this rare type of leukemia is genetically more similar to AML, specific to AML with minimal differentiation.

In conclusion, we show that myeloid gene mutations are enriched in a cohort of ALAL cases strictly diagnosed according to WHO 2016 criteria.

Moreover, our data seem to suggest that double RUNX1 mutations are enriched in leukemias with an undifferentiated phenotype and may support the hypothesis that AUL and AML with minimal differentiation represent a continuum of disease with a similar genetic background. This finding has significant clinical implications, as AUL could be more sensitive to standard AML-oriented therapeutic regimens.

## Data Availability Statement

The datasets presented in this study can be found in online repositories. The names of the repository/repositories and accession number(s) can be found in the article/**Supplementary Material**.

## Ethics Statement

The studies involving human participants were reviewed and approved by Ethics committee of IRCCS Fondazione Policlinico San Matteo, Pavia, Italy. The patients/participants provided their written informed consent to participate in this study.

## Author Contributions

PZ and LA conceived, designed, and supervised the study. GM, MR, AG, SZ, PZ, and LA analyzed and interpreted data and wrote the manuscript. All authors contributed to the article and approved the submitted version.

## Funding

This work was supported by grants of the Fondazione Regionale Ricerca Biomedica (FRRB), Milan, Italy (FRRB project no. 2015-0042, genomic profiling of rare hematologic malignancies, development of personalized medicine strategies, and their implementation into the Rete Ematologica Lombarda clinical network) and by the Italian Ministry of Education, University and Research (MIUR) to the Departments of Molecular Medicine (DMM) of the University of Pavia under the initiative “Dipartimenti di Eccellenza (2018–2022)”. The funder was not involved in the study design, collection, analysis, and interpretation of data, the writing of this article or the decision to submit it for publication.

## Acknowledgments

The authors thank Chiara Chiereghin who helped in the interpretation of data.

## Conflict of Interest

Author ER was employed by the company enGenome srl.

The remaining authors declare that the research was conducted in the absence of any commercial or financial relationships that could be construed as a potential conflict of interest.

## Publisher’s Note

All claims expressed in this article are solely those of the authors and do not necessarily represent those of their affiliated organizations, or those of the publisher, the editors and the reviewers. Any product that may be evaluated in this article, or claim that may be made by its manufacturer, is not guaranteed or endorsed by the publisher.
