# Supplementary material for: Enrichment of Double RUNX1 Mutations in Acute Leukemias of Ambiguous Lineage
Source: Front Oncol. 2021 Aug 31;11:726637. doi: 10.3389/fonc.2021.726637 (PMC8444989; doi:10.3389/fonc.2021.726637)

Supplemental table 1 - On the left the 138 lymphoid-gene panel, on the right the 54 myeloid gene panel.

LYMPHOID-GENE PANEL			MYELOID-GENE PANEL	
AMOTL1	FBXO11	MKI67	ABL1	JAK3
APAF1	FBXW7	MSL2	ASXL1	KDM6A
APC	FGFR3	MYC	ATRX	KIT
ARID1A	FOXO1	MYCBP2	BCOR	KMT2A
ARID3A	GNA13	MYD88	BCORL1	KRAS
ATM	GPR34	NOTCH1	BRAF	MPL
ATP6AP1	GPR98	NOTCH2	CALR	MYD88
ATP6V1B2	GPS2	NRAS	CBL	NOTCH1
B2M	HIST1H1B	P2RY8	CBLB	NPM1
BCL10	HIST1H1C	PCDH10	CBLC	NRAS
BCL2	HIST1H1D	PDE4DIP	CDKN2A	PDGFR1
BCL6	HIST1H1E	PIK3CD	CEBPA	PHF6
BCL7A	HIST1H2AC	PIM1	CSF3R	PTEN
BCL7B	HIST1H2AG	PLA2G4D	CUX1	PTPN11
BCL7C	HIST1H2AM	POU2AF1	DNMT3A	RAD21
BCOR	HIST1H2BE	POU2F2	ETV6/TEL	RUNX1
BIRC3	HIST1H2BI	PTEN	EZH2	SETBP1
BRAF	HIST1H2BK	PTPRD	FBXW7	SF3B1
BTG1	HIST1H3B	REL	FLT3	SMC1A
BTG2	HIST1H3G	RET	GATA1	SMC3
BTK	HLA-A	RRAGC	GATA2	SRSF2
CARD11	HLA-B	SEMA3A	GNAS	STAG2
CCND1	HLA-C	SF3B1	HRAS	TET2
CCND2	HVCN1	SGK1	IDH1	TP53
CCND3	ID3	SIN3A	IDH2	UDAF1
CCR6	IGLL5	SMARCA2	IKZF1	WT1
CD58	IKBKB	SOCS1	JAK2	ZRSR2
CD70	IKZF3	SPEN		
CD79B	IRF2BP2	SPIB		
CDKN2A	IRF4	STAT3		
CHD2	IRF8	STAT6		
CIITA	ITPKB	SWAP70		
CREBBP	KLF2	TBL1XR1		
CTNNB1	KLHL6	TET2		
CXCR4	KMT2C	TNFAIP3		
DDX3X	KMT2D	TNFRSF14		
DTX1	KRAS	TOX		
EBF1	LEF1	TP53		
EGR1	LRP1B	TRAF3		
EGR2	MAP2K1	TRAF7		
EP300	MAP3K14	TRRAP		
ETV6	MAP3K3	USH2A		
EZH2	MAP3K7	VMA21		
FAS	MCRS1	WAC		
FAT1	MEF2B	XBP1		
FAT4	MEF2C	XPO1		

Supplemental table 2 - Clinical features of the patients at diagnosis, chemotherapeutic regimens received, response and allogeneic bone marrow transplant

Patient	Diagnosis (WHO 2016)	Age (y)	Induction CT	Response	Salvage CT	Response2	ABMT
Pt 2	AUL	66	ALL-oriented	NR	AML-oriented	CR	yes
Pt 3	ALAL NOS	71	ALL-oriented	CR	-	-	no
Pt 4	ALAL NOS	46	ALL-oriented	NR	AML-oriented	NR	yes
Pt 5	AUL	72	None	-	-	-	no
Pt 6	AUL	72	None	-	-	-	no
Pt 7	MPAL B/myeloid	52	Lost to follow up	-	-	-	-
Pt 9	MPAL B/myeloid bilinear	63	AML-oriented	CR	-	-	yes
Pt 10	MPAL T/myeloid	45	AML-oriented	CR	-	-	yes
Pt 11	AUL	73	ALL-oriented	CR	-	-	NA
Pt 12	AUL	26	ALL-oriented	CR	-	-	NA

Supplemental Table 3 - Oncogenic and likely oncogenic SNV and small indels per patient

Pt	Gene	Function	Chr	Exon	WT	MT	VAF	C.	P.	Tag
2	ASXL1	frameshift insertion	20	12	-	G	0,50	c.1927dupG	p.G642fs	oncogenic
2	BCOR	stopgain SNV	X	14	C	A	0,07	c.G4810T	p.E1604X	oncogenic
2	EZH2	frameshift deletion	7	10	T	-	0,91	c.1211delA	p.E404fs	oncogenic
2	RUNX1	missense	21	5	A	T	0,43	c.T488A	p.F163Y	oncogenic
2	RUNX1	stopgain SNV	21	7	-	A	0,42	c.777dupT	p.N260_P261delinsX	oncogenic
2	KMT2C	missense	7	14	G	A	0,0452	c.C2291T	p.S764F	oncogenic
2	KMT2D	frameshift deletion	12	31	A	-	0,3125	c.6595delT	p.Y2199fs	oncogenic
3	DNMT3A	missense	2	19	T	A	0,06	c.A2321T	p.E774V	oncogenic
3	DNMT3A	missense	2	23	G	A	0,37	c.C2644T	p.R882C	oncogenic
3	IDH2	missense	15	4	C	T	0,19	c.G419A	p.R140Q	oncogenic
3	NRAS	missense	1	2	C	T	0,18	c.G34A	p.G12S	oncogenic
3	NOTCH1	missense	9	27	A	T	0,1042	c.T5033A	p.L1678Q	oncogenic
4	BCOR	missense	X	8	G	T	0,04	c.C3719A	p.S1240Y	oncogenic
4	ARID1A	missense	1	9	A	G	0,1644	c.A2825G	p.N942S	oncogenic
4	KMT2C	stopgain SNV	7	7	A	C	0,0263	c.T918G	p.Y306X	oncogenic
5	ASXL1	frameshift deletion	20	12	GT	-	0,3	c.2058_2059del	p.686_687del	oncogenic
6	ASXL1	stopgain SNV	20	11	C	T	0,43	c.C1210T	p.R404X	oncogenic
6	FLT3	missense	13	20	T	A	0,08	c.A2504T	p.D835V	oncogenic
6	KRAS	missense	12	2	T	A	0,14	c.A15T	p.K5N	oncogenic
6	NRAS	missense	1	2	C	A	0,04	c.G37T	p.G13C	oncogenic
6	SETBP1	missense	18	4	G	A	0,42	c.G2602A	p.D868N	oncogenic
6	SRSF2	missense	17	1	G	T	0,40	c.C284A	p.P95H	oncogenic
7	IKZF1	missense	7	5	C	T	0,25	c.C484T	p.R162W	oncogenic
7	KDM6A	frameshift deletion	X	23	TATCA	-	0,54	c.3338_3342del	p.1113_1114del	oncogenic
7	NRAS	missense	1	2	C	T	0,46	c.G34A	p.G12S	oncogenic
7	PDE4DIP	missense	1	1	G	T	0,1608	c.C100A	p.Q34K	oncogeni
9	PHF6	missense	X	9	C	G	0,80	c.C932G	p.A311G	oncogenic
9	RUNX1	missense	21	4	T	C	0,37	c.A314G	p.H105R	oncogenic
9	RUNX1	frameshift deletion	21	5	G	-	0,26	c.492delC	p.V164fs	oncogenic
9	SF3B1	missense	2	13	C	T	0,43	c.G1774A	p.E592K	oncogenic
9	FGFR3	missense	4	7	C	T	0,53	c.C899T	p.P300L	oncogenic
10	PHF6	missense	X	10	A	G	0,34	c.A974G	p.Y325C	oncogenic
10	ATM	stopgain SNV	11	51	A	T	0,875	c.A7516T	p.R2506X	oncogenic
11	DNMT3A	missense	2	19	G	A	0,07	c.C2245T	p.R749C	oncogenic
11	NRAS	missense	1	2	C	T	0,05	c.G35A	p.G12D	oncogenic
11	RUNX1	nonsense	21	5	C	A	0,04	c.G427T	p.E143*	oncogenic
11	RUNX1	inframe insertion	21	5	-	TAGCATCTC	0,05	c.442_443insGAGATGCTA	p.A147_T148insRDA	likely oncogenic
11	U2AF1	missense	21	2	G	A	0,07	c.C101T	p.S34F	oncogenic

12	EZH2	missense	7	17	G	A	0,2	c.C1955T	p.S652F	oncogenic
12	PTPN11	missense	12	3	G	A	0,47	c.G181A	p.D61N	oncogenic
12	RUNX1	frameshift insertion	21	5	-	CTCT	0,45	c.382_383insAGAG	p.T128Kfs*11	oncogenic
12	RUNX1	missense	21	5	G	C	0,43	c.C496G	p.R166G	oncogenic
12	U2AF1	missense	21	6	C	T	0,2	c.G467A	p.R156H	oncogenic

Supplemental figure 1 - Median number of mutations in myeloid genes (3, IQR: 1-4) compared to the lymphoid ones (1, IQR: 0-1) (Wilcoxon matched-pairs signed-rank test p=0.012)

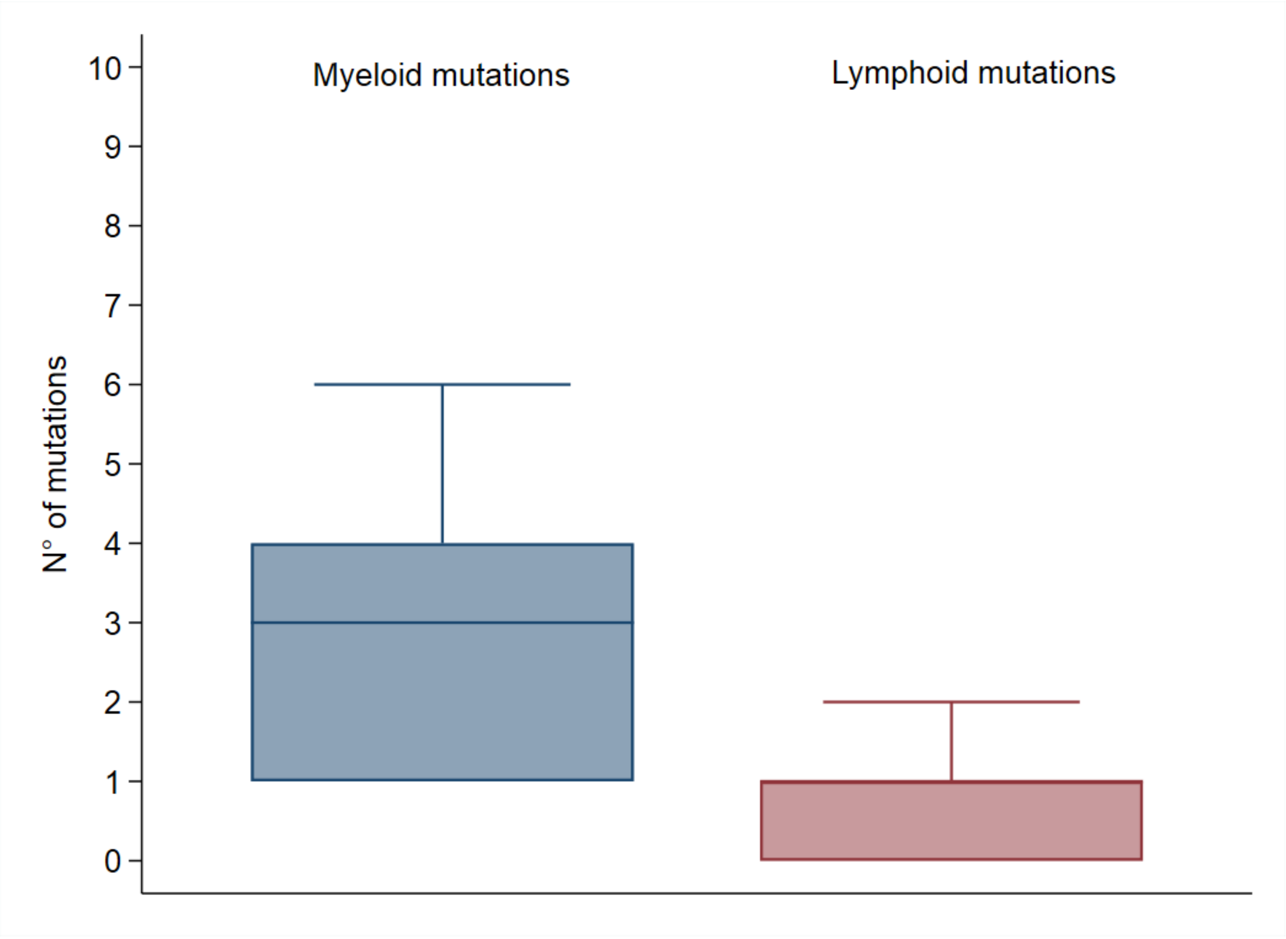

Supplemental Figure 2 - RUNX1 c.488T>A variant oncogenic mechanism in patient 2. The nucleotide mutation is highlighted in green. RUNX1 exon 5 (NM_001754) is highlighted in yellow, introns are not highlighted. The sequence in the box represents the canonical splice site where, in normal conditions, the splicing machinery cuts the DNA. The sequence underscored within exon 5 represents the cryptic splice site region.

The splicing predictive score of the canonical splice site is high (Fruitfly 0,99), compared with the wild type sequence of the cryptic splice site region (Fruitfly 0,47). However, the presence of the c.488T>A variant markedly increases the prediction score of the cryptic splice site sequence (Fruitfly 1,00). According to this prediction, RNA reads obtained from patient 2 show the presence of a 23-nucleotide deletion (c.486-508del23). Therefore, we hypothesize that the oncogenic mechanism resides in the erroneous recognition of the cryptic splice site sequence by the splicing machinery, thus resulting in the cut of the last 23 nucleotides of exon 5. This non-canonical splice site recognition ultimately induces a shift in the reading frame predicted to cause a truncated RUNX1 protein (p.F163EfsX41).

ATCATTGAATATACATTTAATTTTAGAATAATCACTACACAAATGCCCTAAAAGTGTATG
TATAACATCCCTGATGTCTGCATTTGTCCTTTGACTGGTGTTTAGGTGGTGGCCCTAG
GGGATGTTCCAGATGGCACTCTGGTCACTGTGATGGCTGGCAATGATGAAAACTACT
CGGCTGAGCTGAGAAATGCTACCGCAGCCATGAAGAACCAGGTTGCAAGATTTAATG
ACCTC**AGGTA**TGTCGGTCGAAGTGGAAGAGGTACGTTATCTGTCAAAACTATGCTTG
AAACACGTTTCATGGCAACAAACCCACATTTCAAAATTCTAGTGATTTCTGTGATGGAA
TCCCTAGAAACTCGGTCTGTCTTAACATTCTTATTGCTATGTCAAGCTCTTA

Supplemental Figure 3 - Circos plot representing gene pathways and mutations in ALAL subtypes

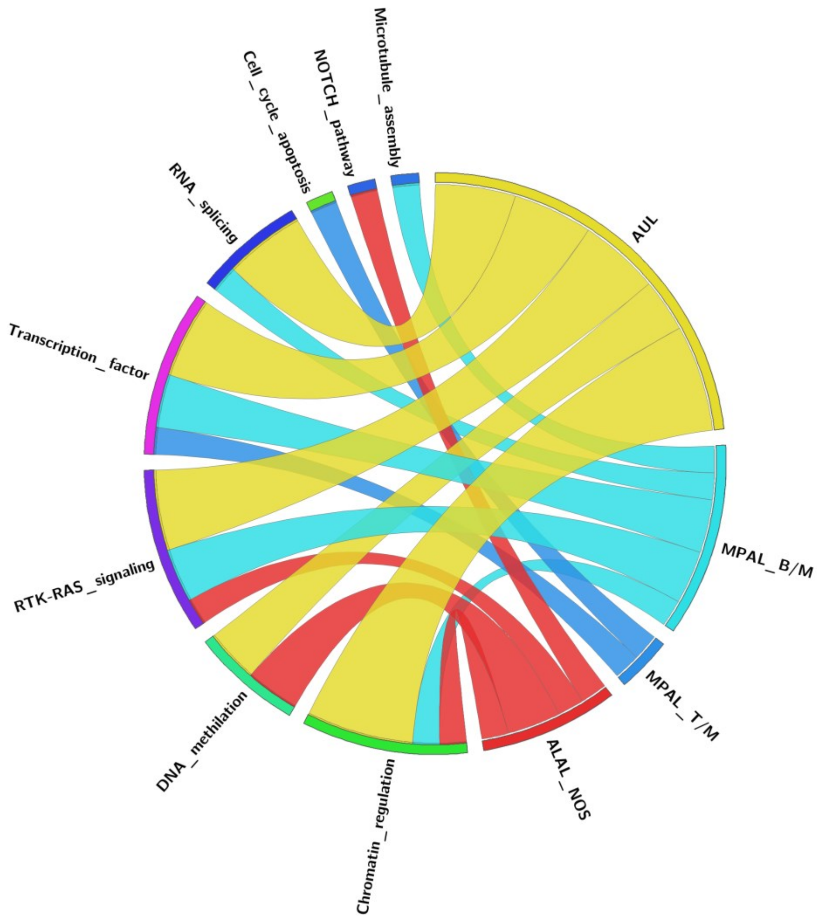

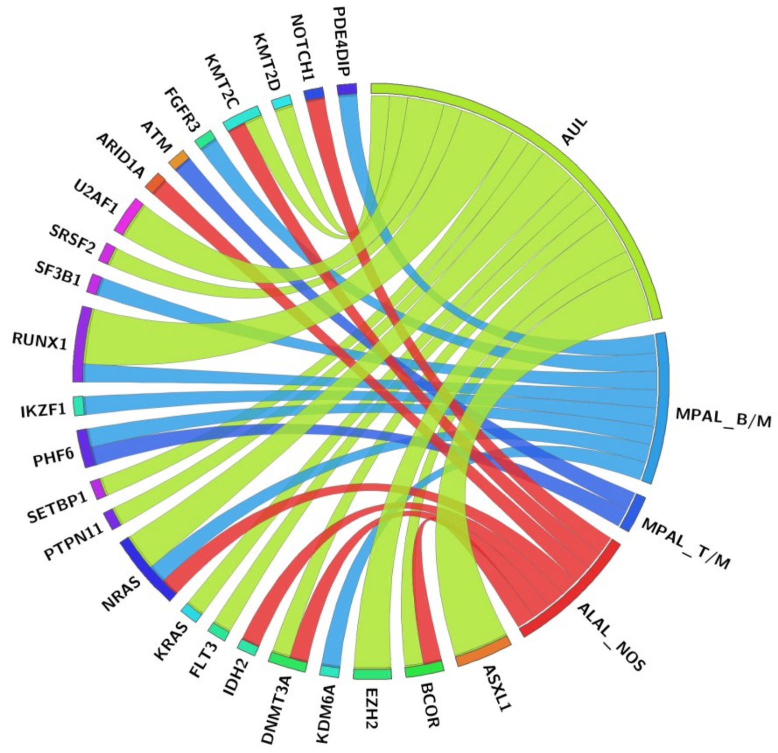

Supplement: Supplementary file 1 [file DataSheet_1.pdf]
